# Supplementary figures and images for: Beyond a PPR-RNA recognition code: Many aspects matter for the multi-targeting properties of RNA editing factor PPR56
Source: PLoS Genet. 2023 Aug 21;19(8):e1010733. doi: 10.1371/journal.pgen.1010733 (PMC10482289; doi:10.1371/journal.pgen.1010733)

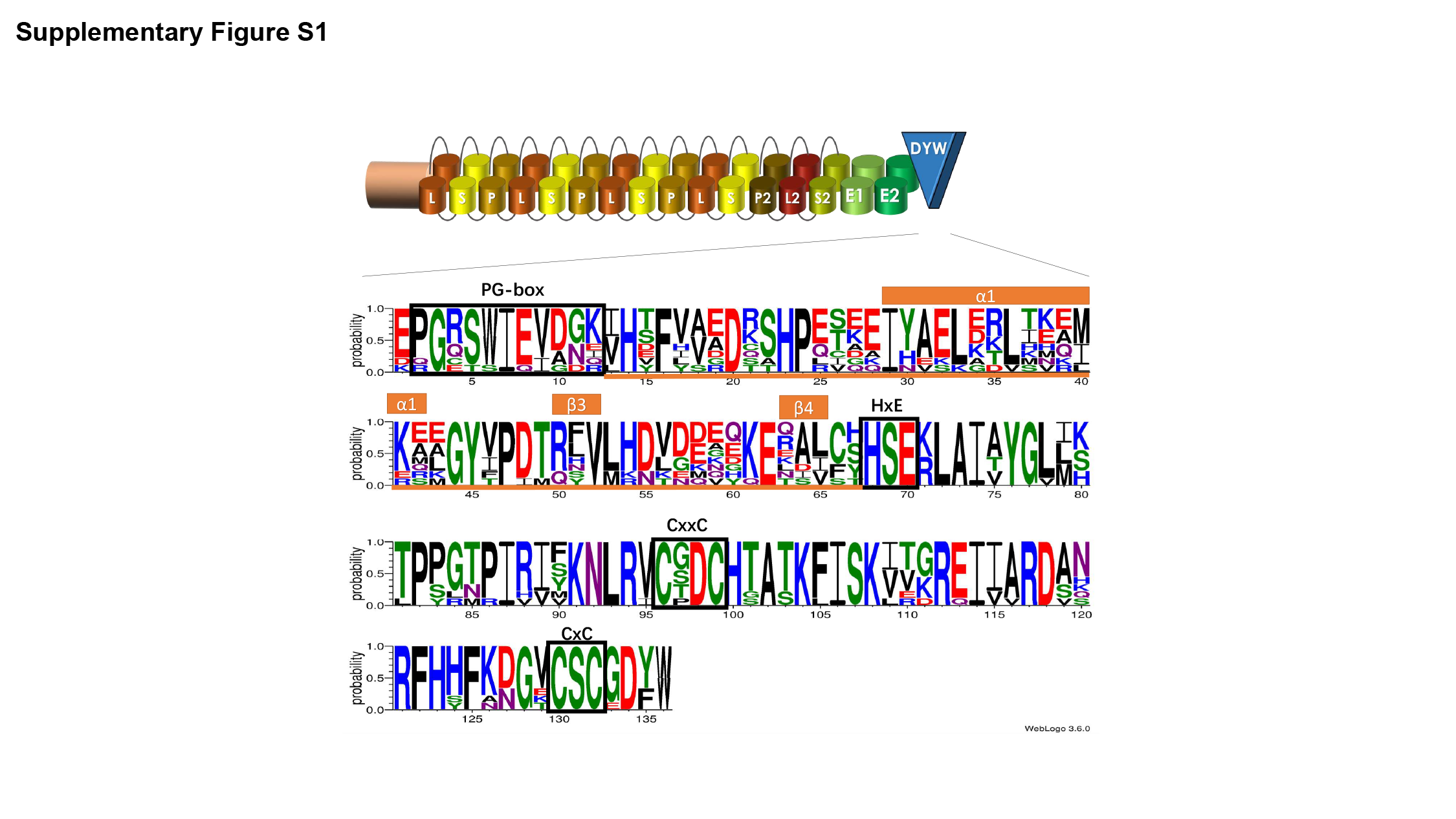

Supplement: S1 Fig — The conservation plot based on the alignment of the DYW domains of nine functionally characterized RNA editing factors of Physcomitrium patens has been obtained with WebLogo [89]. Highlighted with frames are the characteristic PG box at the N-terminus of the DYW domain, the signature motifs for coordination of two zinc ions including the catalytic center (HSE) of the cytidine deaminase and the region of amino acids 37–42 discussed as relevant for compatibility for creating protein chimeras [49]. The “gating domain” as recently defined from X-ray structural analysis after crystallization of the OTP86 DYW domain [46] is highlighted in orange. Several residues have been selected for the study of mutants (Fig 1B). (TIF) [file pgen.1010733.s001.tif]

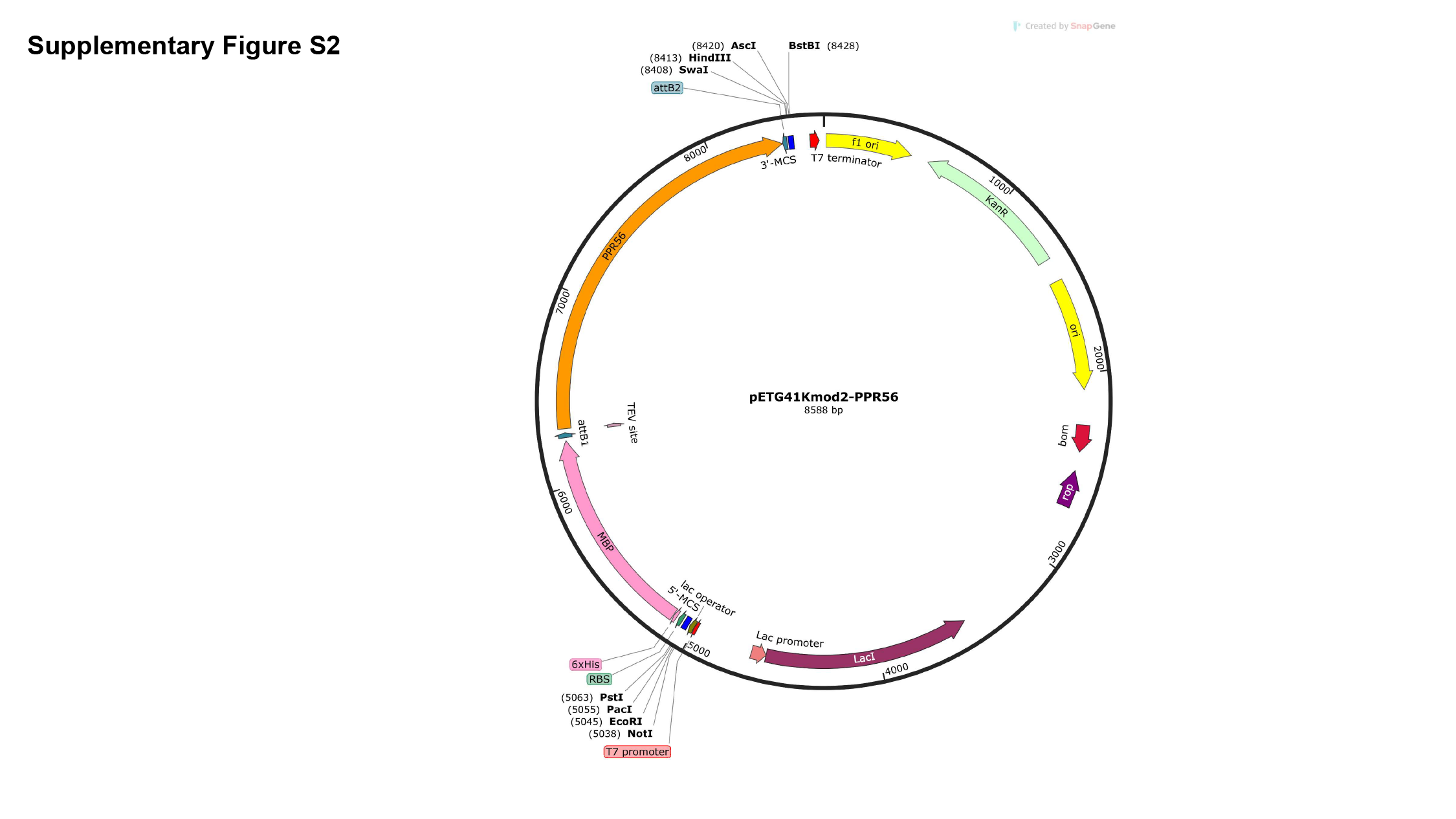

Supplement: S2 Fig — Vector pET41Kmod for expression of RNA editing factors and their targets has been reported previously [36]. Coding sequences of RNA editing factors are inserted by Gateway cloning resulting in flanking attachment attB sequences connecting in-frame via a TEV cleavage site to the upstream maltose binding protein (MBP) and an N-terminal His6 tag. Transcription is driven from a T7 promoter controlled by a lac operator and translation is initiated by a ribosome binding site (RBS). PPR56 is cloned with an N-terminal extension of 14 native amino acids upstream from its N-terminal PPR L-14. Target sequences were designed with hybridized oligonucleotides inserted by classic cloning into a multiple cloning site (MCS, SwaI-HindIII-AscI-BstBI) in the 3’-UTR between attB2 and a T7 terminator. A new vector variant pET41Kmod2 has been created which also allows for cloning target sequences alternatively upstream into the 5’-UTR in a second MCS (NotI-EcoRI-PacI-PstI) inserted into a previous XbaI site. The vector map was created with SnapGene Viewer 6.2.1 (https://www.snapgene.com). (TIF) [file pgen.1010733.s002.tif]

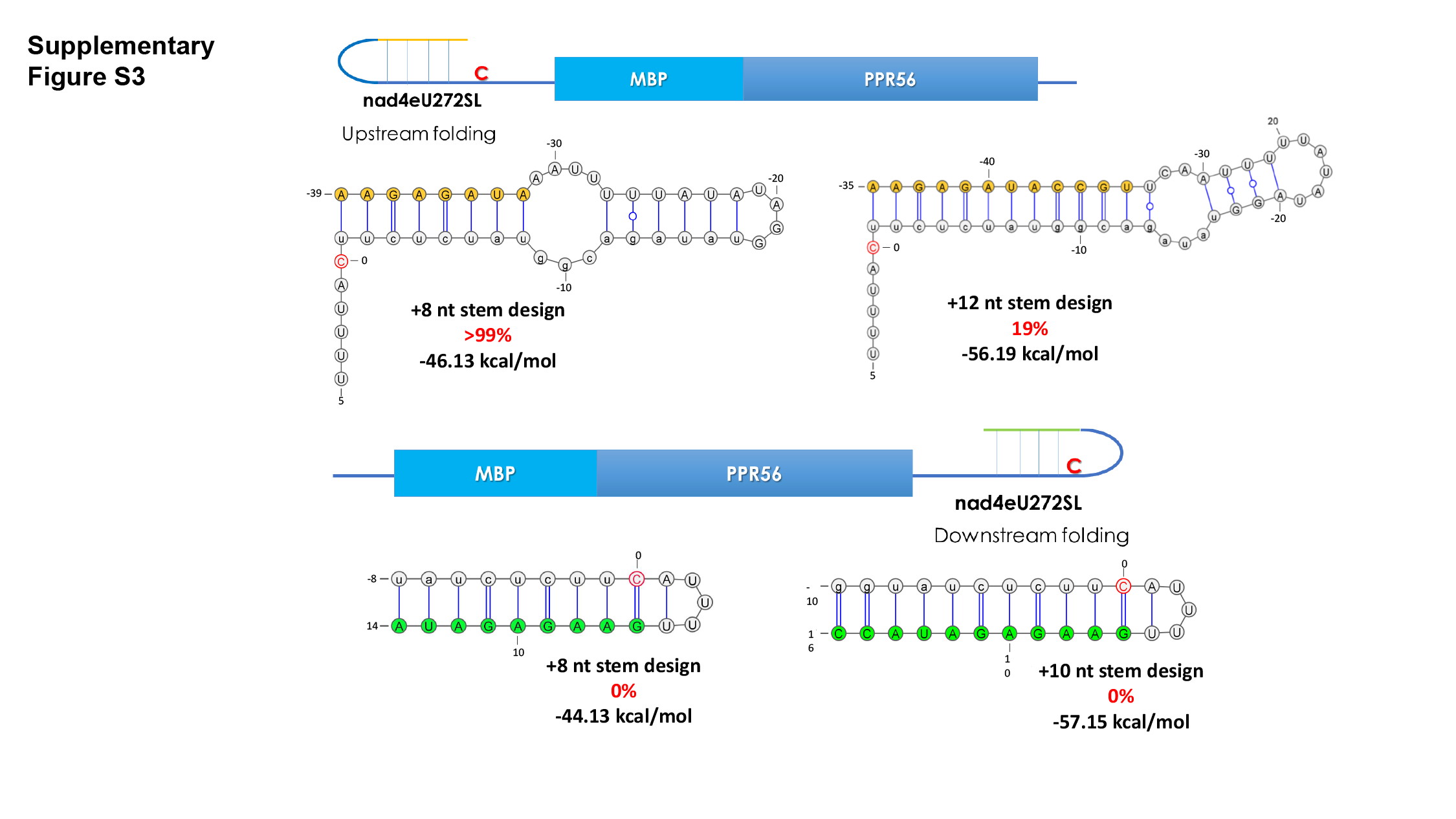

Supplement: S3 Fig — Artificial sequences have been added upstream (yellow) or downstream (green) to embed the cytidine targeted for RNA editing (red) into secondary structures. The sequence upstream of the cytidine editing target that is supposedly juxtaposed with the PPR array of PPR56 (see Fig 1A) is shown in small letters. The RNAfold WebServer of the ViennaRNA package [90] was used to predict the secondary structures. RNA structure models were created with VARNAv3-93 (https://varna.lri.fr). (TIF) [file pgen.1010733.s003.tif]

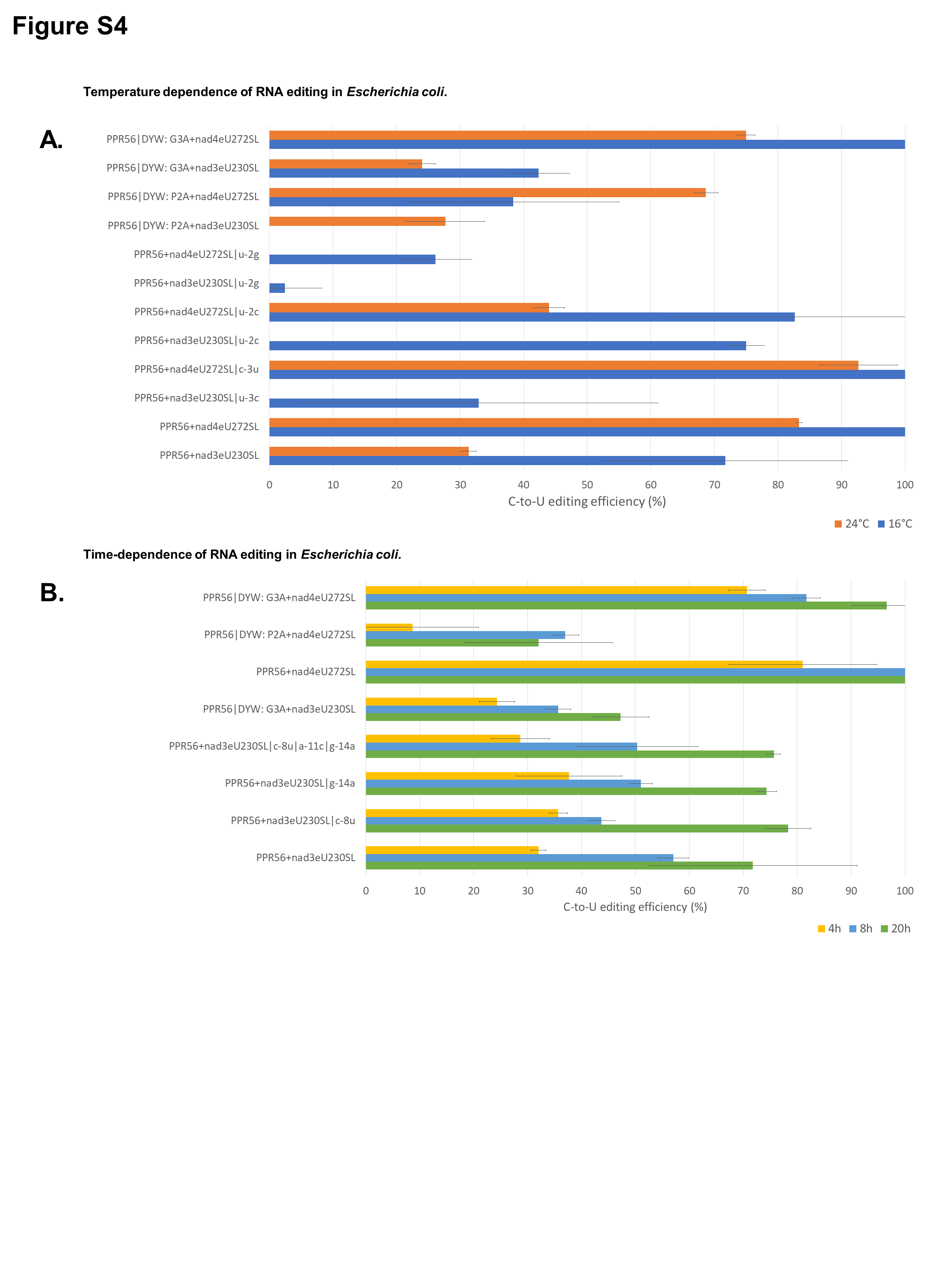

Supplement: S4 Fig — A. RNA editing was checked at an elevated temperature of 24° (orange bars) instead of the routinely used 16°C (blue bars) for heterologous protein expression in the E. coli Rosetta 2 (DE3) arctic express system for a selection of altogether twelve constructs. The elevated temperature of 24°C generally disfavors RNA editing compared to incubation at 16° both on nad4 and on nad3 targets with the interesting exception of the PPR56|DYW:P2A mutant. B. RNA editing was checked for eight selected constructs also at shorter incubation times of only 4 h or 8 h, respectively, instead of the routinely used 20 h of incubation at 16°C after induction of expression. A reduction of RNA editing is seen in all cases of shorter incubation times except for the efficiently edited nad4 target, which already shows >99% editing after 8 h of incubation. (TIF) [file pgen.1010733.s004.tif]
