# Supplementary material for: Beyond a PPR-RNA recognition code: Many aspects matter for the multi-targeting properties of RNA editing factor PPR56
Source: PLoS Genet. 2023 Aug 21;19(8):e1010733. doi: 10.1371/journal.pgen.1010733 (PMC10482289; doi:10.1371/journal.pgen.1010733)
Supplement: Attachment — Submitted filename: Yang-etal-PPR56-response-to-reviews-final_20230706.pdf [file pgen.1010733.s009.pdf]

Dear editors @ PLoS Genetics, dear Dr. Qu and Dr. Tan,

Thank you very much for providing the three favorable reports on our manuscript and the constructive suggestions for amendments. We have accordingly made requested additions, cloned and analyzed several further recombinant constructs and changed text and figures, as we will outline below in detail with our numbered responses in bold font. The line numbers in the latter refer to the correction-tracked version of the manuscript revision, which we provide in parallel.

Dear Drs. Knoop and Schallenberg-Rüdinger,

Thank you very much for submitting your Research Article entitled 'Beyond a PPR-RNA recognition code: Many aspects matter for the multi-targeting properties of RNA editing factor PPR56' to PLOS Genetics. The manuscript was fully evaluated at the editorial level and by three independent peer reviewers. The reviewers appreciated the attention to an important topic but identified some concerns that we ask you address in a revised manuscript. We therefore ask you to modify the manuscript according to the review recommendations. Your revisions should address the specific points made by each reviewer. In addition, we ask that you:

1) Provide a detailed list of your responses to the review comments and a description of the changes you have made in the manuscript.

2) Upload a Striking Image with a corresponding caption to accompany your manuscript if one is available (either a new image or an existing one from within your manuscript). If this image is judged to be suitable, it may be featured on our website. Images should ideally be high resolution, eye-catching, single panel square images. For examples, please browse our archive. If your image is from someone other than yourself, please ensure that the artist has read and agreed to the terms and conditions of the Creative Commons Attribution License. Note: we cannot publish copyrighted images.

We hope to receive your revised manuscript within the next 30 days. If you anticipate any delay in its return, we would ask you to let us know the expected resubmission date by email to plosgenetics@plos.org.

If present, accompanying reviewer attachments should be included with this email; please notify the journal office if any appear to be missing. They will also be available for download from the link below. You can use this link to log into the system when you are ready to submit a revised version, having first consulted our Submission Checklist.

While revising your submission, please upload your figure files to the Preflight Analysis and Conversion Engine (PACE) digital diagnostic tool. PACE helps ensure that figures meet PLOS requirements. To use PACE, you must first register as a user. Then, login and navigate to the UPLOAD tab, where you will find detailed instructions on how to use the tool. If you encounter any issues or have any questions when using PACE, please email us at figures@plos.org.

Please be aware that our data availability policy requires that all numerical data underlying graphs or summary statistics are included with the submission, and you will need to provide this upon resubmission if not already present. In addition, we do not permit the inclusion of phrases such as "data not shown" or "unpublished results" in manuscripts. All points should be backed up by data provided with the submission.

To enhance the reproducibility of your results, we recommend that you deposit your laboratory protocols in protocols.io, where a protocol can be assigned its own identifier (DOI) such that it can be cited independently in the future. Additionally, PLOS ONE offers an option to publish peer-reviewed

clinical study protocols. Read more information on sharing protocols at https://plos.org/protocols?utm_medium=editorial-email&utm_source=authorletters&utm_campaign=protocols

Please review your reference list to ensure that it is complete and correct. If you have cited papers that have been retracted, please include the rationale for doing so in the manuscript text, or remove these references and replace them with relevant current references. Any changes to the reference list should be mentioned in the rebuttal letter that accompanies your revised manuscript. If you need to cite a retracted article, indicate the article's retracted status in the References list and also include a citation and full reference for the retraction notice.

PLOS has incorporated Similarity Check, powered by iThenticate, into its journal-wide submission system in order to screen submitted content for originality before publication. Each PLOS journal undertakes screening on a proportion of submitted articles. You will be contacted if needed following the screening process.

To resubmit, you will need to go to the link below and 'Revise Submission' in the 'Submissions Needing Revision' folder. Please let us know if you have any questions while making these revisions.

Yours sincerely, Bao-Cai Tan, Guest Editor, PLOS Genetics; Li-Jia Qu, Section Editor, PLOS Genetics

Dear Drs. Knoop and Schallenberg-Rüdinger,

Thanks for submitting your manuscript to PLoS Genetics.

This manuscript by Yang et al. used a heterologous system in E. coli to dissect the determinants in the P. patens PPR56 recognition of its targets by introducing various mutations to both the RNA and the PPR protein. The findings, as pointed by the three reviewers in this field, have a significant contribution to the understanding of the molecular basis of RNA metabolism / RNA editing. Although all the reviewers recognize the high quality and value of this work, they also made some comments and suggestions, which I think could improve this work. In particular,

1) if the authors could analyze function of the E1 and E2 motif in target site recognition, it will provide a complete story.

Response 0.1: OK. See our detailed response below. Altogether, we tried to keep a reasonable balance between the remarks on already showing too much data and requests for yet further additions by the reviewers. Given that the first author Yingying Yang (YY), after finishing her PhD in Bonn, is presently returning to her home country China, we have decided to put the requested additional cloning (altogether eight additional new constructs) and experimental work into the hands of Jingchan Xie (JX), a master student who was previously supervised by YY. Accordingly, JX now became a further co-author.

2) Also, it would strengthen the conclusion if the authors could test the mutation u-16c that improves the conceptual fit to PPR S-13NS.

Response 0.2: OK, we agree that this is a valuable addition to the mutant set. Please, see our detailed response 1.1 below.

3) Reviewers also suggested a revision of the text, to make it more clear, logical, and easier to the reader.

Response 0.3: OK. We rearranged the introduction to enhance background information and a historic perspective as requested by reviewers #2 and #3 (see detailed response 3.2).
Given reviewer #1's remarks that "The paper is well written, easy to read…" we were, however, careful in not introducing too many further text changes. All introduced text changes are outlined in detail below.

4) The parts focusing on "Exploring novel candidate targets" in the Results and Discussion parts are not entirely clear. This should be explained in detail.

Response 0.4: Please see our detailed response 3.5 below.

Considering these comments and suggestions, a minor revision of this manuscript is required before it can be accepted for publication in PLoS Genetics.

Reviewer #1: In this report, the authors tested the RNA editing efficiency of the mutations of moss DYW-type RNA editing factor PPR56 on its target variants in the heterologous bacterial system. They found that (1) the mutations in DYW domain of PPR56 show different effects on the editing of two native targets; (2) the two native targets of PPR56 display different nucleotides opposite of its three central L-type PPR motifs; (3) several transcript features beyond the region ultimately targeted by the PLS-type PPR array likely contribute to enhance the editing activity. RNA secondary structures inhibit RNA editing, whereas native sequences further upstream enhance RNA editing. This work expands our understanding of how PPR proteins recognize and edit substrate RNA targets. The paper is well written, easy to read with experiments supporting most of the conclusions. I have only minor comments.

Response 1.0: We highly appreciate the reviewer's friendly remarks and positive evaluation of our manuscript and the constructive suggestions that we addressed as detailed below.

1. The N-terminal PPR motifs of PPR65 appear to have a significant impact on the editing of nad3eU230SL target, as the mutation u-15c abolishes editing completely. It would be nice if the authors test the mutation u-16c that improves the conceptual fit to PPR S-13NS. Increased editing efficiency is a stronger support for the conclusion.

Response 1.1: OK, a very good point, although we do not *necessarily* expect improved editing for matching changes in the mostly variable N-terminal PPRs. In any case, we have now created two additional single mutants addressing position -16 not only for the nad3 target (nad3eU230SL|u-16c) but also for the nad4 target (nad4eU272SL|a-16c) and also for an additional double mutant nad3eU230SL|u-16c|c-6u. The three new constructs have been added into a new version of figure 2 and the corresponding text addition under results (line 225 ff.) reads:
"A cytidine would be expected to best match PPR S-13NS. Accordingly, we also included mutants nad4eU272SL|a-16c and nad3eU230SL|u-16c and the double target mutant nad3eU230SL|u-16c|c-6u in our collection of mutant variants. For the conceptual improvement of the matches opposite of PPR S-13NS we do observe moderate decreases in editing efficiencies to 77% for the nad4eU272SL target and to 68% for nad3eU230SL, in line with the finding that N-terminal PPRs of editing frequently do not match their target nucleotides following the PPR-RNA code. However, the double target mutant nad3eU230SL|u-16c|c-6u adding the favorable c-6u exchange still performed very well with an editing efficiency of 94% (Fig. 2)."

2. It is not clear from the Materials and methods whether the transit peptide was removed from the proteins assayed in bacterial experiments.

Response 1.2: OK. This information had unfortunately been given too late under M&M only in the context to protein mutant variants. We have rearranged the text accordingly, with the second sentence of M&M (line 622 ff.) now starting "PPR56 coding sequences (lacking the N-terminus with signal peptide and including only 14 amino acids upstream of the first clearly identified PPR)…"

3. The authors should explain why the corresponding triple-mutations converting positions -14, -11 and -8 to the identities in the respective other target decrease editing at the nad4 target to 26% but improve editing at the nad3 target to 76%.

Response 1.3: A somewhat surprising outcome, indeed. We have added the following statement (lines 304 ff.): "This, at first glance surprising, outcome may indicate that in some cases the concept of one-PPR-to-one-nucleotide matches may be too simplified and that certain successions of PPRs or target nucleotides could be disfavored for PPR-RNA interactions."

4. In Fig.8, a negative control should be included to avoid that the reason for the increase in editing efficiency at nad3eU230SL site is due to the addition of a nucleic acid sequence before the target.

Response 1.4: A good point and a very interesting result, as it turned out!
To address this issue raised by the reviewer, we have created a further construct creating an entirely artificial sequence with all transitions to the former upstream *nad4* target. Quite surprisingly, this is also enhancing editing at the downstream *nad3* target. We have added this new construct to an updated version of figure 8 and addressed it with additional text (lines 412 ff.) as follows:
"Surprisingly, however, we also observed an enhancing effect on RNA editing efficiency of the downstream *nad3* target to 93% when an artificial sequence introducing all possible transitions (a-g and c-u) into the upstream nad4 sequence was used (Fig. 8). Evidently, the sensitivity of editing the *nad3* target is not only reflected by changes in the PPR56 protein or in the target sequence but also by placement of the latter in the wider transcript environment as further confirmed below."

Consequently, we have also toned down or deleted statements on editing factor enrichment or scanning later at the end of this paragraph (lines 420 ff.) and elsewhere (lines 130 ff. and 562 ff.)

5. P492. "of" should be "or".

Response 1.5: Corrected, thank you.

Reviewer #2 [uploaded review]: In this manuscript, Yang et al provide what could arguably be considered the most extensive study to date of the interaction between the cis and trans recognition elements in plant organellar RNA editing. Using a clever in bacteria system they finally realize what the pioneering teams that worked in this field from the early 1990's to circa 2010 had only dreamt of: dissecting both the RNA portion (cis element) surrounding the cytosine to be edited and the PPR protein (trans factor) recognizing it. The manuscript contains numerous experiments, which are (mostly) clearly presented. The various claims from the authors are well supported by their results. I however have some minor concerns about the way some of the experiments are presented and I also wonder whether the organization of the manuscript could be modified to keep the progression more logical. This is indeed a long and complex manuscript to read, even for somebody with a good knowledge of the field. I personally think some experiments could be left out and even become a story of their own (the off target analysis) without diminishing the overall quality or impact of the work presented here. Finally, I think the analysis of the E1 and E2 motif is sorely lacking here, preventing the work to be considered complete or exhaustive.

Response 2.0: We appreciate the overall very favorable remarks by the reviewer. As stated above in our general responses we tried to avoid larger rearrangements of the text given that this could cause conflicts with the assessments of reviewers #1 and #3. In particular, we prefer to retain the data already presented in the originally submitted version (including the off-target analyses), as we strongly assume that this makes our points stronger (see below) and taking them out now would likely be not in-line with ideas of the other reviewers.

More specifically, here my comments:

Authors should provide more detailed about the way they calculate editing efficiency. I assume it is from peak area measurements in Sanger sequencing RT-PCR products (indicated in the legend of the Supp table 1) but it should be made explicit in the material and methods (referring to a previous manuscript is not enough). The Supp table1 should be carefully checked and revised as I could spot inconsistencies within it. For example line 5 shows 100% editing with a 0 sd for the editing site nad3eU230SL at 16°C while suppl figure 4A and all the results in the manuscripts show and talk about an editing efficiency >70%. Another weird result is displayed at the line 55 of the table where the sd indicated is 74, an inconsistent result according to the efficiencies calculated on the same line. I did not go through the entire table in details but these 2 discrepancies indicate that the table should be carefully revised and verified.

Response 2.1: We have added the following text under Materials and Methods (line 651 ff.): "RT-PCR sequencing chromatograms were analyzed with MEGA 7 [82] and Bioedit 7.0.5.3 [83]. RNA editing was quantified by the ratio of the thymidine peak to the sum of thymidine and cytidine peaks in the editing position."
Also, we apologize for inconsistencies in supplementary table 1, which have been repaired. In the text, we have consistently avoided indicating "100%" of RNA editing as this is not meaningful for a post-transcriptional process.

Also, authors claim they made 3 biological replicates (reported in the sup table 1) but they did not explain what they meant by biological replicates. Are they independent E coli transformations? Independent colonies coming from a single transformation and grown at the same moment? At different moments? I also wonder why authors did not provide statistical analyses to verify if the differences in editing efficiencies they identified were significant or not. This was for example done by Ichinose et al. 2022, communication biology (ref 54 in the ms).

Response 2.2: In line with the above point, we have also clarified this issue with further text in line 652 ff. that reads: "RNA editing was routinely checked for three biological replicates, i.e. three independent bacterial clones after re-transformation of a given plasmid construct after control sequencing." We prefer to avoid an arbitrary threshold for statistical significance but rather focus the attention on cases of most dramatic mutant effects.

Another point that is only slightly addressed in the math and meth, once again referring to a former publication, is the verification of the various biases that can influence the observed editing efficiencies. Conceptually, it can be influenced by both the expression level of the protein and the quantity of the RNA target. One possibility is that the effects observed could be the consequence of an influence of the introduced mutation on the abundance/stability of the protein/RNA. SDS PAGE (with western blots?) and qRT-PCR could be displayed to convince the reader that the effects observed are indeed a direct consequence on the editing kinetic itself.

Response 2.3: Admittedly, any heterologous setup may certainly affect results, but this even holds true for *in planta* complementation assays where strong overexpression is mostly used to complement mutants of the naturally very lowly expressed editing factors (with about only one

protein copy per mitochondrion (Fuchs et al. 2020). Certainly, background effects cannot be excluded completely (see below points 3.4 and 3.5) but we stressed the congruent results so far obtained from bacterial and human heterologous setups, which make a significant contribution of other factors very unlikely.

As mentioned by the authors citing ref 36 and 38, it is now clear that the E1 and E2 domain are also involved in RNA recognition and binding and this has also been studied in a similar in cellulo setup in ref 54. I think the authors should have tried to introduce mutations in the E1 and E2 motifs in addition to the corresponding -1 to -4 nt of the RNA editing site. In its current state, the manuscript is really convincing for all but the 3-4 nt immediately upstream of the editing site and 2 PPR like domains of the proteins. If authors do that, their study would be complete.

Response 2.4: OK. Although this point rather adds further content and we would rather think that detailed investigations of E domains would be much more "a story of its own" than our off-target analyses, we have nevertheless added two mutant constructs following the suggestion of this reviewer. These are added to figure 1 as a new panel C and are now addressed with the following additional text section in lines 198 ff.: "The precise role of the TPR-(tetratricopeptide repeat) like E1 and E2 motifs linking the DYW domain and the upstream PPR arrays of plant RNA editing factors is still unclear. Given their location in the proteins and the distant similarity of TPRs to PPRs, E1 and E2 may contribute to binding to nucleotide positions -3 and -2 upstream of the cytidine target, but with a matching code different from the one for the P- and S-type PPRs. In the E2 motifs of RNA editing factors, the corresponding positions '5' and 'L' (i.e. position 34 in the TPR-like E motifs) are strongly dominated by valine (V) and lysine (K), respectively, suggesting a structural rather than nucleotide-specific role. In contrast, position '5' of E1 shows no significant conservation at all, but position 'L' (34) of the E1 motif has a resemblance to PPRs with aspartate (D) or asparagine (N) dominating in the conservation profiles. Given the unique opportunity to test impacts on the two different targets of PPR56 we created mutants PPR56|E1:N34D and PPR56|E2:K34A and tested them on both targets (Fig. 1C). While mutant PPR56|E1:N34D could be expected to now favor the *nad3* target given presence of a uridine in position -3, we did not see a significant change of editing efficiency at either target. In stark contrast, editing efficiencies dropped dramatically for mutant PPR56|E2:K34A to only 7% on the nad4eU272SL target and abolished RNA editing altogether at the nad3eU230SL target, again confirming the overall higher sensitivity of the latter."

The corresponding new figure legend 1C reads: "Positions 34 ('last') of the E1 and E2 motifs potentially juxtaposed with nucleotide positions -3 and -2 upstream of the edited cytidine have been mutated and tested on the two native targets of PPR56 with RNA editing remaining unaffected by the PPR56|E1:N34D mutant, but dropping dramatically for the PPR56|E2:K34A mutant."

In line, we have now added E1 and E2 amino acid identities '5' and 'L' in figure 1A and added a sentence in the corresponding legend, reading "The corresponding amino acid identities in the TPR-like E1 and E2 motifs are indicated in italics". Furthermore, we added "… and upstream E motifs" in the results headline (line 159)

I don't see the point of the paragraph starting line 281 about the creation of START and STOP codons. It is of potential high interest in a biotech point of view, putatively allowing using this tool as a mean to control gene expression post transcriptionally but it doesn't give any additional information about the recognition mechanisms that are being investigated here.

Response 2.5: We do not quite agree on this point and believe this short paragraph fits well into considerations on positions in the immediate environment – and this all the more so now with the requested addendums on the E1 and E2 domains! Moreover, this particular aspect is related to

other ongoing work and our communication with other laboratories including unpublished work on other editing factors, including synthetic designer PPRs. We still wish to make readers aware that an editing factor like PPR56 is generally flexible enough to allow for addressing start and stop editing targets. Given that the text section extends over only six lines in the manuscript, we prefer to leave it in.

Similarly, I wonder if the off target analysis is really necessary to the manuscript. Don't get me wrong, it is of high quality and value but I think it could become a story of its own. In any case, if kept in the manuscript, I strongly believe it should be moved towards the end and after the experiments where duplicated/triplicated cis recognition elements are tested. This would make the reading more logical, directly putting the duplication experiment after the observations showing that distal element outside of the -20 region could influence editing efficiency.

If maintained, the off target analysis should be made clearer. The RNA-Seq part is for example quite difficult to understand in its design. I think a table summarizing the datasets used would solve most of the problem. In the current state of the manuscript, the reader has to go into the sup table 2 to try to understand and identify the different data used. The summary table could contain part of the info in the legend tab of the suppl table but also an clear indication about which data have been compared to which ones. I had to spend a long time to try to make sense out of it and although I think the work is good, I don't think it is presented in a way that facilitates its understanding. The data should also be me made publicly accessible. One thing authors did not do in this part is to try the opposite strategy: Here they use RNA-Seq data to look for off targets editing sites and then use the new sites to define a consensus motif. A complementary analysis would be to do something similar to what the authors did to identify cox3eU290SF and look for potential ediding sites in E.coli based on sequence and then they could compare to the consensus motif they identify through RNA-Seq.

Response 2.6: Please see the above point. We do not quite agree on this issue. In fact, rather than including an investigation of the E domains, we still consider the extended off-target analyses a central part of the story presented here. Very important in that regard is comparing the off-target consensus profile to the data obtained from our mutant experiments.
The reason for presenting off-targets in figure 7 (and not at the very end) was that we first need to *identify* off-targets before they could be tested for performance in the tandem target arrangements shown in figures 8 and 10.
An accession for data deposition is meantime available and we have added that under M&M (line 681 f.): "Primary data have been deposited at the NCBI under BioProject accession number PRJNA984633"

Finally, I want to highlight how pleasant it is to read such a study as it is a perfect example of how science advances. Basically, none of the results presented in the manuscript are surprising (this is not a critic!), most if not all of them are reminiscent of earlier approaches using in vitro and in organello system. I think the authors could have acknowledged this fact more in the discussion, the so far only mention the in vitro work from the Takenaka/Brennicke group showing that cis element duplication improved editing efficiency. They could have done the same with works showing distal effect (outside of the reduced cis element) both in vitro and in organello, works that dissected nucleotides by nucleotides the cis recognition elements and showed that not all the positions had the same importance in the recognition, or that nucleotides immediately adjacent to editing site were crucial in some sites but not all of them. All the beautiful work presented here is in line with what has been already obtained with more archaic methods, therefore demonstrating how robust these strategies were and how knowledge slowly increases, step by step.

Response 2.7: Again, thank you for the friendly general remarks.
Following the advice, we have now cited ten further older publications (new references 5-14 in line 40) in the context of the explanatory addendum to the introduction that was requested by referee #3, starting in line 37 (see our detailed response 3.2 below).

Reviewer #3: The expression of genes in plant organelles requires coordination of multiple RNA processing steps at the post-transcriptional level. This is run by a multitude of nucleus-encoded RNA-binding proteins (RBPs) that control RNA stability, processing, and degradation. PPR proteins play a pivotal role in these critical processing events in land plant mitochondria. In their MS, Yingying Yang et al analyzed the PPR56 factor from Physcomitrium patens in RNA editing. The authors provide with further details, following their progress made by using a heterologous system for RNA editing in the bacterium E. coli system, regarding the RNA editing properties of PPR56 to its two native transcripts in P. patens, nad3eU230SL and nad4eU272SL. Taking the advantage of this powerful system, the authors introduces various mutations to both the RNA binding PPR module, as well as to the deaminase (DYW) domain, to study the basis of RNA recognition and C-to-U substitutions mediated by the PPR56 cofactor. Undoubtedly, the MS and data therein are of high quality and provides with an excellent contribution to our understanding of these important cellular processes. Noteworthy, the authors are also leading experts in these field, where they have a significant contribution to the understanding of the molecular basis of RNA metabolism / RNA editing and the roles played by nuclear-encoded organellar-localized factors in mitochondrial (or plastidial) RNA maturation. I mostly have minor comments to the authors.

Response 3.0: We are grateful for the kind words and the positive overall assessment of our paper by reviewer #3.

Specific comments:

1. The text may benefit from some revisions to make the MS, data and points raised therein easier to understand and read.

Response 3.1: We hope that the text changes now introduced in response to the detailed remarks by the three reviewers as detailed above and below have enhanced clarity.

2. Introduction part: Although referred later in this part, it would be easier for the reader if the authors provide with some details about PPR proteins as modular RBPs (often site-specific) as well to their "extra" related domains (e.g., E, E+, DYW) that are associated with substrate RNA "editing to the beginning of the Intro.

Response 3.2: OK, we agree. To address this remark, we have added a new text section in the introduction, directly following an addition in response to a remark by reviewer #2, see point 2.7 above. The new text section starting in line 37 reads:
"Among other insights, very early functional studies on plant RNA editing based on *in organello, in vitro* or transplastomic studies had already demonstrated that the specificity for identifying cytidine targets largely resides in their immediate sequence environment, mainly within circa 20 upstream nucleotides [5–14]. The molecular characterization of the *trans*-acting specificity factors, however, ultimately relied on reverse genetic approaches leading to the identification of CRR4 as a first identified chloroplast and MEF1 as the first mitochondrial RNA editing factor in *Arabidopsis thaliana* [15,16]. Such site-specific editing factors feature sequence-specific RNA-binding pentatricopeptide repeats (PPRs) of the so-called PLS-type followed by "extension" domains E1 and E2 plus a C-terminal DYW cytidine deaminase, which may alternatively be supplied *in trans*. PLS-

type PPR arrays typical for RNA editing factors are characterized by “long” and “short” PPR variants with characteristic consensus profiles along with the canonical P-type PPRs of 35 amino acids. The E1 and E2 motifs of 34 amino acids are distantly related to TPRs (Tetratricopeptide Repeats) existing in other proteins, where they have been shown to mediate protein-protein rather than protein-RNA interactions.”

3. Mutating the PPR and DYW domains: One way to identify and characterize a domain is to find the part of a target protein that has sequence or structural similarities with a template through homology alignment. This is nicely demonstrated in the seq alignment of the nine DYW domains of the P. patens (Fig. S1) but should be also presented by a phylogenetic illustration as well (i.e., how close are these to one another? And what variation exist in other DYW domains in mosses as well as other species). An interesting question is whether swapping different DYW domains (e.g. from PPR65 into PPR56) may influences the editing, assuming that the sequence specificity resides mainly within the PPR repeats…

Response 3.3: Phylogenetic trees of the *Physcomitrium patens* RNA editing factors have been published more than once (e.g. Oldenkott et al 2020) and we prefer to not duplicate this here. As expected, orthologues are clearly discernible for closely related mosses and more paralogues can be identified by wider taxon sampling. The latter, however, also does not impact the conservation profile as shown. This will also be addressed in another manuscript from our group currently under revision, which also treats the creation of protein chimeras, swapping DYW domains (or parts thereof) of different editing factors.

4. Regarding the effects of aa's substitutions on editing activity: This is done in a heterologous system and thus the effects should be taken with some cautious. Also, the fact that an amino acid was found to be conserved at the nine DYW domains in P. patens doesn't necessarily indicate that it has any importance for the activity or folding of the protein/domain (the phylogenetic analysis may make this more prevalent). The authors may want to comment on possible effects (predictions) of these substitutions on the folding of the DYW domain or the PPR arrays.

Response 3.4: We can certainly not fully exclude that single amino acid exchanges like the ones reported here could have far-ranging impacts on the overall structure of the protein. We consider such dramatic overall changes quite unlikely, and in particular for the changes replacing one against another “code” amino acids in PPR positions 5 and L as shown in figure 3. Most importantly, we consider reliable predictions on such large-scale structure changes not feasible. Even powerful tools like the modern AlphaFold algorithm will not be able to model protein structure in interaction with an RNA, which is of fundamental relevance here for a potentially different “induced fit” of PPR56 to its two different targets.

5. The parts focusing on “Exploring novel candidate targets” in the Results and Discussion parts are not entirely clear. The expression, folding and activity of the recombinant P.s.PPR56 in E. coli can affect its specificity and activity resulting with various off-targets in E. coli that may not occur under the native condition in the plant mitochondria.

Response 3.5: We can certainly not be 100% sure, that the targets would also be edited in plant mitochondria, but high similarity of addressed targets of PPR56 in the different genetic systems now investigated (plant mitochondria, *E.coli* and also human cells (Lesch et al. 2022)) confirms that binding behaviour of the protein is not affected strongly by the different genetic environments. Certainly, the influence of other proteins on PPR56 cannot be fully excluded, but to our knowledge the only clear evidence for proteins affecting the match of PPRs to an RNA are the cases of MORFs/RIPs (Takenaka et al. 2012, Bentolila et al. 2012). These, however, are clearly absent both in *Physcomitrium patens* and in *Escherichia coli* or in human cells (Gutmann et al. 2020).

6. Intro line 47: To a large part, the complex editosomes of angiosperms. Please elaborate on (plant mito) editosome(s).

Response 3.6: OK, in line with other reviewers' remarks, we have rearranged the introduction providing more background information, also from a historical perspective (see lines 37 ff.). Addressing specifically this point we have also added in line 55 ff.: "Aside from the core RNA editing factors for target recognition, the complex angiosperm editosome protein assemblies, for example, feature MORFs/RIPs or ORRMs [3,18–20]."

7. Intro lines 56-57: "Physcomitrium patens has a prominent role with its only 13 C-to-U RNA editing sites assigned to nine site-specific RNA editing factors. Please provide with REFs.

Response 3.7: OK. References [3,21,22,33] have been added here at the end of that sentence (now line 72).

8. Line 63: To readers which are not familiar with PPRs, please explain what are PLS-PPRs.

Response 3.8: OK, this is now done in the reshaped introduction (lines 44 ff.)

9. Lines 111-115: This part is unclear and thus could be simplified.

Response 3.9: OK. In line with point 1.4 above, the sentence is now shortened after "… transcript environment." (now line 130)

10. Results line 118: " a typical "complete", and likely evolutionarily ancestral," looks to me as an unnecessary comment.

Response 3.10: OK. Deleted now (new line number 135).

11. Line 137: The coding sequence of PPR56 is cloned in fusion with…, consider in-frame to

Response 3.11: OK. Changed as suggested (now line 153).

12. Line 406: Exploring novel candidate targets – "It is important to keep in mind that orthologues of a functionally characterized". That's probably depends on the homology, and these might rather represent paralogs in such cases?!

Response 3.12: We do not quite agree here. A phylogenetically clearly defined orthologue in another species may well address another editing target in that species which is simply not present but given as a pre-edited thymidine in other taxa.

13. The discussion part can be shortened and more focused on the data (see comments 3 to 5).

Response 3.13: We can't quite see how the requested extra considerations on the above points could possibly shorten the discussion. Moreover, we are afraid that the reviewers assessment on weighting different issues are quite different and would prefer not to delete any text elements that the respective other reviewers found informative and important. However, we did delete some text speculating on editing factor scanning or enrichment, most notably as a consequence of the additional data obtained in response to the suggestion by reviewer #1 (see point 1.4)

14. Also, and with regard to lines 537-541 is PPR56 is expected to be part of an editosome in P. patens, and whether this factor could be associated with additional RBPs in E. coli?! How these situations influence the activities seen in vivo, in vitro or in the heterologous E.coli systems?

Response 3.14: Please see our response above on point 3.5

Again, we wish to express our gratitude for the many constructive suggestions by the three reviewers. We hope that we have succeeded in addressing all points adequately.

With kind regards, also on behalf of the other co-authors,

Volker Knoop, Yingying Yang and Mareike Schallenberg-Rüdinger
