# Supplementary material for: Beyond a PPR-RNA recognition code: Many aspects matter for the multi-targeting properties of RNA editing factor PPR56
Source: PLoS Genet. 2023 Aug 21;19(8):e1010733. doi: 10.1371/journal.pgen.1010733 (PMC10482289; doi:10.1371/journal.pgen.1010733)
Supplement: Attachment — Submitted filename: Yang_et_al.docx [file pgen.1010733.s008.docx]

In this manuscript, Yang et al provide what could arguably be considered the most extensive study to date of the interaction between the *cis* and *trans* recognition elements in plant organellar RNA editing. Using a clever in bacteria system they finally realize what the pioneering teams that worked in this field from the early 1990’s to circa 2010 had only dreamt of: dissecting both the RNA portion (cis element) surrounding the cytosine to be edited and the PPR protein (*trans* factor) recognizing it. The manuscript contains numerous experiments, which are (mostly) clearly presented. The various claims from the authors are well supported by their results. I however have some minor concerns about the way some of the experiments are presented and I also wonder whether the organization of the manuscript could be modified to keep the progression more logical. This is indeed a long and complex manuscript to read, even for somebody with a good knowledge of the field. I personally think some experiments could be left out and even become a story of their own (the off target analysis) without diminishing the overall quality or impact of the work presented here. Finally, I think the analysis of the E1 and E2 motif is sorely lacking here, preventing the work to be considered complete or exhaustive. More specifically, here my comments:

Authors should provide more detailed about the way they calculate editing efficiency. I assume it is from peak area measurements in Sanger sequencing RT-PCR products (indicated in the legend of the Supp table 1) but it should be made explicit in the material and methods (referring to a previous manuscript is not enough). The Supp table1 should be carefully checked and revised as I could spot inconsistencies within it. For example line 5 shows 100% editing with a 0 sd for the editing site nad3eU230SL at 16°C while suppl figure 4A and all the results in the manuscripts show and talk about an editing efficiency >70%. Another weird result is displayed at the line 55 of the table where the sd indicated is 74, an inconsistent result according to the efficiencies calculated on the same line. I did not go through the entire table in details but these 2 discrepancies indicate that the table should be carefully revised and verified.

Also, authors claim they made 3 biological replicates (reported in the sup table 1) but they did not explain what they meant by biological replicates. Are they independent E coli transformations? Independent colonies coming from a single transformation and grown at the same moment? At different moments? I also wonder why authors did not provide statistical analyses to verify if the differences in editing efficiencies they identified were significant or not. This was for example done by Ichinose et al. 2022, communication biology (ref 54 in the ms).

Another point that is only slightly addressed in the math and meth, once again referring to a former publication, is the verification of the various biases that can influence the observed editing efficiencies. Conceptually, it can be influenced by both the expression level of the protein and the quantity of the RNA target. One possibility is that the effects observed could be the consequence of an influence of the introduced mutation on the abundance/stability of the protein/RNA. SDS PAGE (with western blots?) and qRT-PCR could be displayed to convince the reader that the effects observed are indeed a direct consequence on the editing kinetic itself.

As mentioned by the authors citing ref 36 and 38, it is now clear that the E1 and E2 domain are also involved in RNA recognition and binding and this has also been studied in a similar *in cellulo* setup in ref 54. I think the authors should have tried to introduce mutations in the E1 and E2 motifs in addition to the corresponding -1 to -4 nt of the RNA editing site. In its current state, the manuscript is really convincing for all but the 3-4 nt immediately upstream of the editing site and 2 PPR like domains of the proteins. If authors do that, their study would be complete.

I don’t see the point of the paragraph starting line 281 about the creation of START and STOP codons. It is of potential high interest in a biotech point of view, putatively allowing using this tool as a mean to control gene expression post transcriptionally but it doesn’t give any additional information about the recognition mechanisms that are being investigated here.

Similarly, I wonder if the off target analysis is really necessary to the manuscript. Don’t get me wrong, it is of high quality and value but I think it could become a story of its own. In any case, if kept in the manuscript, I strongly believe it should be moved towards the end and after the experiments where duplicated/triplicated cis recognition elements are tested. This would make the reading more logical, directly putting the duplication experiment after the observations showing that distal element outside of the -20 region could influence editing efficiency.

If maintained, the off target analysis should be made clearer. The RNA-Seq part is for example quite difficult to understand in its design. I think a table summarizing the datasets used would solve most of the problem. In the current state of the manuscript, the reader has to go into the sup table 2 to try to understand and identify the different data used. The summary table could contain part of the info in the legend tab of the suppl table but also an clear indication about which data have been compared to which ones. I had to spend a long time to try to make sense out of it and although I think the work is good, I don’t think it is presented in a way that facilitates its understanding. The data should also be me made publicly accessible. One thing authors did not do in this part is to try the opposite strategy: Here they use RNA-Seq data to look for off targets editing sites and then use the new sites to define a consensus motif. A complementary analysis would be to do something similar to what the authors did to identify cox3eU290SF and look for potential ediding sites in E.coli based on sequence and then they could compare to the consensus motif they identify through RNA-Seq.

Finally, I want to highlight how pleasant it is to read such a study as it is a perfect example of how science advances. Basically, none of the results presented in the manuscript are surprising (this is not a critic!), most if not all of them are reminiscent of earlier approaches using *in vitro* and in *organello* system. I think the authors could have acknowledged this fact more in the discussion, the so far only mention the in vitro work from the Takenaka/Brennicke group showing that cis element duplication improved editing efficiency. They could have done the same with works showing distal effect (outside of the reduced cis element) both *in vitro* and *in organello*, works that dissected nucleotides by nucleotides the cis recognition elements and showed that not all the positions had the same importance in the recognition, or that nucleotides immediately adjacent to editing site were crucial in some sites but not all of them. All the beautiful work presented here is in line with what has been already obtained with more archaic methods, therefore demonstrating how robust these strategies were and how knowledge slowly increases, step by step.
